# Retinal Complications in a Patient With Pediatric Thalassemia: A Case Report

**DOI:** 10.7759/cureus.86610

**Published:** 2025-06-23

**Authors:** Zineb Hilali, Oumaima El Korno, Saad Benchekroun Belabbes, Samira Tachfouti, Lala Ouafae Cherkaoui

**Affiliations:** 1 Faculty of Medicine, Hôpital des Specialités de Rabat, Rabat, MAR; 2 Ophthalmology, Ibn Sina Hospital, Rabat, MAR; 3 Ophthalmology A, Ibn Sina Hospital, Rabat, MAR; 4 Ophthalmology, Hôpital des Specialités de Rabat, Rabat, MAR

**Keywords:** beta-thalassemia major, blood transfusion-related toxicity, macular hemorrhage, ocular complications, pediatric ophthalmology, retinal involvement

## Abstract

The authors report a case of retinal involvement in a pediatric patient with beta-thalassemia major, highlighting the importance of an early ophthalmologic assessment.

A 10-year-old male with a known diagnosis of beta-thalassemia major presented with complaints of decreased visual acuity. A comprehensive ophthalmologic examination, including fundus evaluation and fluorescein angiography, was performed. The fundoscopic examination revealed a macular hemorrhage and marked vascular tortuosity. Imaging supported the presence of retinal hypoperfusion without signs of neovascularization. The retinal findings were attributed to chronic anemia and iron overload related to repeated blood transfusions.

Retinal abnormalities can occur in patients with beta-thalassemia major due to systemic factors such as anemia and iron toxicity. Early detection through routine ophthalmologic screening is essential to prevent potentially irreversible visual impairment in this patient population.

## Introduction

Thalassemia is a hereditary hemoglobinopathy characterized by defective synthesis of globin chains. Beta-thalassemia major requires lifelong transfusions, leading to complications such as iron overload [1]. While systemic manifestations are well-documented, ocular involvement is underreported. This case highlights retinal changes in a child with beta-thalassemia and discusses potential mechanisms and management.

## Case presentation

A 10-year-old boy with a known history of beta-thalassemia major presented to the ophthalmology clinic with complaints of blurred vision in the right eye for two weeks. His medical history included regular blood transfusions since infancy and chelation therapy with deferoxamine.

**Figure 1:**
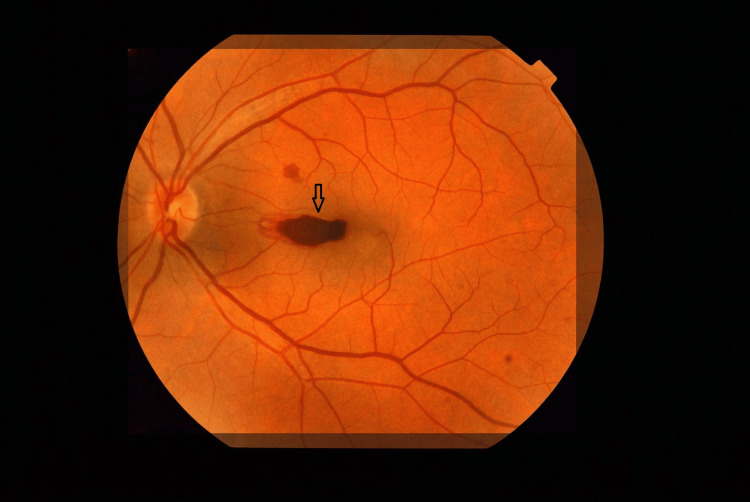
A well-defined macular hemorrhage is visible (black arrow), irregular in shape, and located centrally within the macula.

On ocular examination, the patient's visual acuity was measured at count fingers in the left eye and 20/20 in the right eye. The anterior segments were normal in both eyes, showing no signs of inflammation. Fundus examination of the right eye revealed a macular hemorrhage accompanied by surrounding retinal pigment epithelium changes (Figure 1).

**Figure 2:**
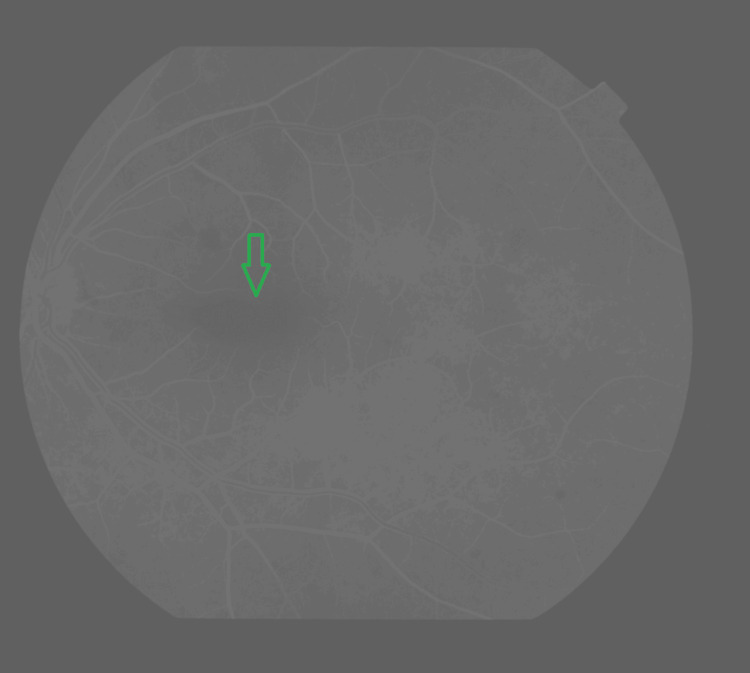
Angiography revealing macular hypofluorescence corresponding to the hemorrhagic spot (green arrow), with no areas of ischemia or neovascularization.

Fluorescein angiography of the posterior pole revealed generally preserved retinal vascular architecture, with clear delineation of both arterial and venous branches. In the macular region, there was a distinct hypofluorescent area consistent with a blockage effect, likely due to intraretinal or subretinal hemorrhage (Figure 2). No signs of capillary dropout, neovascularization, or late-phase leakage are identified, which argues against proliferative retinopathy or active inflammatory processes at this stage. The optic disc demonstrated normal perfusion without evidence of hyperfluorescence, leakage, or staining. 

From a systemic perspective, the patient exhibited clinical signs consistent with chronic anemia and significant iron overload, likely a consequence of ongoing transfusion therapy. Evidence also pointed to mild hepatic stress, which may be attributed to iron deposition or the potential side effects of chelation treatment.

The patient’s chelation therapy was reviewed and optimized. A follow-up with ophthalmology was scheduled every three months to monitor the hemorrhage resolution. No surgical intervention was deemed necessary at this stage.

## Discussion

In patients with thalassemia, retinal abnormalities are not uncommon and result from a complex interplay of disease-related factors and treatment effects. Chronic anemia, a hallmark of thalassemia, leads to sustained tissue hypoxia [2]. The retina, being one of the most metabolically active tissues in the body, is particularly vulnerable to oxygen deprivation. This can manifest as retinal ischemia, with subsequent hemorrhages and microvascular changes that threaten visual integrity [3-4].

In addition to anemia, repeated blood transfusions often result in systemic iron overload [5]. Without effective chelation therapy, excess iron accumulates in various tissues, including the retina, where it exerts toxic effects through oxidative stress and damage to the retinal pigment epithelium (RPE). This toxicity may be subtle initially but can contribute to progressive retinal degeneration over time [6].

Iron chelation, while essential in managing overload, carries its own risks. Deferoxamine, a commonly used chelating agent, has been associated with ocular side effects, particularly when used at high doses or over extended periods [7]. These include changes to the RPE, visual field defects, and even night blindness [8]. However, in the present case, the patient had not exceeded the toxic thresholds of deferoxamine, reducing the likelihood of drug-induced retinal damage [9-10].

In our case, the patient presented with retinal hemorrhages, a known complication in thalassemia. These lesions can cause transient or permanent visual disturbances, depending on their location, especially if the macula is involved, and the extent of hemorrhage. The angiographic findings align with retinal hemorrhagic changes, likely secondary to underlying systemic factors, such as chronic anemia and vascular fragility, common in patients with thalassemia [10]. The absence of neovascular activity or widespread ischemia is reassuring.

In such cases, early ophthalmologic evaluation and supportive management are critical. This includes stabilizing hemoglobin levels through transfusion and reassessing iron chelation protocols to minimize further retinal injury [11].

Ultimately, a multidisciplinary approach is essential to preserve visual function in thalassemia patients, with regular monitoring, prompt recognition of retinal changes, and tailored adjustments in treatment strategies [12].

## Conclusions

This case underscores the importance of routine ophthalmologic screening in pediatric thalassemia patients. Retinal changes, while often subtle initially, can lead to significant visual deficits if undetected. Multidisciplinary care is vital to ensure systemic and ocular health in these children.
